# Increasing Spectrum in Antimicrobial Resistance of *Shigella* Isolates in Bangladesh: Resistance to Azithromycin and Ceftriaxone and Decreased Susceptibility to Ciprofloxacin

**Published:** 2007-06

**Authors:** Rahman Mahbubur, Shereen Shoma, Harunur Rashid, Shams El Arifeen, A.H. Baqui, A.K. Siddique, G.B. Nair, D.A. Sack

**Affiliations:** 1 ICDDR,B, GPO Box 128, Dhaka 1000, Bangladesh; 2 Johns Hopkins University Bloomberg School of Public Health, 615 N. Wolfe St., Baltimore, MD 21205, USA

**Keywords:** Azithromycin, Ciprofloxacin, Drug resistance, Microbial, E-test, Microbial sensitivity tests, Nalidixic acid, R-plasmid, Shigella, Bangladesh

## Abstract

Antimicrobial resistance of *Shigella* isolates in Bangladesh, during 2001-2002, was studied and compared with that of 1991-1992 to identify the changes in resistance patterns and trends. A significant increase in resistance to trimethoprim-sulphamethoxazole (from 52% to 72%, p<0.01) and nalidixic acid (from 19% to 51%, p<0.01) was detected. High, but unchanged, resistance to tetracycline, ampicillin, and chloramphenicol, low resistance to mecillinam (resistance 3%, intermediate 3%), and to emergence of resistance to azithromycin (resistance 16%, intermediate 62%) and ceftriaxone/cefixime (2%) were detected in 2001-2002. Of 266 recent isolates, 63% were resistant to ≥3 anti-*Shigella* drugs (multidrug-resistant [MDR]) compared to 52% of 369 strains (p<0.007) in 1991-1992. Of 154 isolates tested by E-test in 2001-2002, 71% were nalidixic acid-resistant (minimum inhibitory concentration [MIC] ≥32 μg/mL) and had 10-fold higher MIC_90_ (0.25 μg/mL) to ciprofloxacin than that of nalidixic acid-susceptible strains exhibiting decreased ciprofloxacin susceptibility, which were detected as ciprofloxacin-susceptible and nalidixic acid-resistant by the disc-diffusion method. These strains were frequently associated with MDR traits. High modal MICs were observed to azithromycin (MIC 6 μg/mL) and nalidixic acid (MIC 128 μg/mL) and low to ceftriaxone (MIC 0.023 μg/mL). Conjugative R-plasmids-encoded extended-spectrum ß-lactamase was responsible for resistance to ceftriaxone/cefixime. The growing antimicrobial resistance of *Shigella* is worrying and mandates monitoring of resistance. Pivmecillinam or ciprofloxacin might be considered for treating shigellosis with caution.

## INTRODUCTION

Shigellosis is one of the significant causes of diarrhoeal diseases in the developing world. Worldwide, an estimated 165 million cases and 1.1 million deaths (mostly in developing countries) occur annually (1–4). Antimicrobial therapy has been recommended for patients with shigellosis because it can limit the clinical course of illness and reduce the risk of complications and the duration of faecal excretion of the causative organism, reducing the spread of infection (2–5). The therapy also improves the growth and nutritional status of affected children, especially in developing countries (5). A major problem, however, is the increasing resistance of *Shigella* spp. to useful antimicrobial agents (3,6–11).

Over the decades, *Shigella* isolates resistant to multiple agents, such as sulphonamides, tetracycline, ampicillin, trimethoprim-sulphamethoxazole (SXT), and nalidixic acid have been reported from many countries, including Bangladesh (3,6–11), resulting in difficulties in the selection of empirical therapy. However, newer antimicrobial agents, such as mecillinam, ciprofloxacin, ceftriaxone, cefixime, and azithromycin, were found to be effective in treating multidrug-resistant *Shigella*-associated infections (6,12–14). Recently, *Shigella* strains resistant to ciprofloxacin have emerged in Bangladesh and India (8,15–16).

Currently, we do not have sufficient information on the susceptibility patterns of *Shigella* isolates to many useful antimicrobial agents, such as mecillinam, ciprofloxacin, levofloxacin, azithromycin, ceftriaxone, and cefixime. Given the impact of suboptimal use of antimicrobial agents in Bangladesh and the ability of *Shigella* to develop resistance after the introduction of new antimicrobial agents for treatment, it is not unlikely that antimicrobial resistance patterns in Bangladesh have changed since they were reported in the 1990s (11). Thus, the continuing changing patterns of resistance of *Shigella* isolates indicate the need for monitoring antimicrobial susceptibility. We, therefore, investigated the trends in antimicrobial resistance of *Shigella* isolates and compared these with previous data to suggest timely recommendations for empirical antimicrobial therapy, if needed.

## MATERIALS AND METHODS

### Clinical samples and bacterial strains

The sources of faecal samples were diarrhoeal patients who attended the Dhaka Hospital of ICDDR,B and whose clinical conditions required stool cultures in a microbiology laboratory as suggested by physicians during the study period. All relevant laboratory data were archived. We analyzed resistance to useful antimicrobial agents of 369 (8%) of 4,597 *Shigella* strains isolated in the Clinical Laboratory of ICDDR,B, which were collected monthly (32-33 strains per month) during 1991-1992. To compare data on antimicrobial resistance with those of 1991-1992, we studied 266 (8%, 22-23 strains monthly) of 3,337 *Shigella* isolates cultured during 2001-2002.

### Microbiological techniques

Faecal samples were cultured onto MacConkey and Salmonella-*Shigella* agars for the isolation of *Shigella* spp., which were identified biochemically by the standard methods (17) and grouped serologically by slide agglutination with specific antisera (Denka Saiken, Tokyo, Japan). Each isolate was tested for susceptibility to ampicillin, chloramphenicol, trimethoprim-sulphamethoxazole, tetracycline, nalidixic acid, ciprofloxacin, mecillinam, ceftriaxone, and gentamicin by the Kirby-Bauer disc-diffusion method in 1991-1992 (11,18). Antimicrobial susceptibility was determined by the Clinical Laboratory Standards Institute (CLSI; formerly NCCLS) guidelines using Muller-Hinton agar, commercial antimicrobial discs (Oxoid, Basingstoke, United Kingdom), and Escherichia coli (ATCC 25922) as reference strain (18). During 2001-2002, isolates were tested to an additional eight antimicrobial agents: erythromycin, azithromycin, amoxicillin-clavulanate, cephalothin, streptomycin, rifampicin, moxifloxacin, and levofloxacin. MICs of nalidixic acid, ciprofloxacin, levofloxacin, ceftriaxone, and azithromycin were determined by E-test (AB Biodisk, Solna, Sweden) for a subset of *Shigella* isolates during 2001-2002.

Production of extended-spectrum ß-lactamase (ESBL) was detected by the double-disc synergy test (DDST) using cefotaxime (30 μg), ceftriaxone (30 μg), and ceftazidime (30 μg) discs placed 20 mm (centre to centre) from the amoxicillin (20 μg)/clavulanate (10 μg) disc, and an increase in diameter of zone of inhibition by the synergy of clavulanate indicated production of ESBL (19).

### Transfer of ceftriaxone resistance plasmid by conjugation

Conjugation between ceftriaxone-resistant *Shigella* isolates (donor) and Escherichia coli K12 strain (F-, lac^-^, Nal^R^) was performed according to the method of Neu et al. (20). Transconjugants were selected on MacConkey agar containing ceftriaxone (6 μg/mL) that produce lactose-fermenting pink colonies in contrast to non-lactose-fermenting pale colonies of *Shigella*. All putative transconjugants were examined for antimicrobial susceptibility and plasmid profiles to obtain transconjugants. They were tested for production of ESBL (18). All transconjugants were cultured twice on MacConkey agar plate containing ceftriaxone (6 μg/mL) to exclude contamination with donors. In the second transfer of R-plasmid by conjugation, ceftriaxone resistance and production of ESBL were transferred from transconjugants E. coli to ceftriaxone-susceptible and trimethoprim-sulphamethoxazole-resistant wild *Shigella sonnei*. The second-generation *Shigella* transconjugants were selected on MHA plates supplemented with ceftriaxone (6 μg/mL) and trimethoprim (24 μg/mL) and were tested for antimicrobial susceptibility, ESBL, and R-plasmid.

### Plasmid analysis

Plasmid DNA was extracted from four ceftriaxone-resistant *Shigella* isolates and all transconjugants according to the method of Portnoy et al. (21) and was separated by electrophoresis in 0.5% agarose gel, stained, and visualized. Reference plasmid markers—V517 and 39R861—were used for determining the size of unknown plasmids.

### Statistical analysis

The significance of differences in the proportions of antimicrobial resistance and of the relative prevalence of each *Shigella* species was determined by the chi-square test. Two-tailed tests were applied.

## RESULTS

Of 266 of *Shigella* isolates tested for antimicrobial susceptibilities by the disc-diffusion method in 2001-2002, *S. flexneri* (51%) was the predominant species, followed by *S. boydii*, *S. sonnei*, and *S. dysenteriae* (Table 1). Overall, *Shigella* isolates had high rates of resistance to tetracycline (79%), trimethoprim-sulphamethoxazole (72%), ampicillin (56%), nalidixic acid (51%), and chloramphenicol (42%). Moderate-to-low rate of resistance to azithromycin (16%), gentamicin (4%), mecillinam (3%), and third-generation cephalosporins (TGC), such as cefixime (2%) and ceftriaxone (2%) was observed. Many isolates were intermediate to azithromycin (62%), amoxicillin-clavulanate (26%), ciprofloxacin (12%), and mecillinam (3%). No resistance to levofloxacin and moxifloxacin was detected. Resistance to azithromycin and third-generation cephalosporins was detected for the first time among recent (2001–2002) isolates in our study. None of the *Shigella* isolates had complete resistance to ciprofloxacin.

**Table 1:** Antimicrobial susceptibility results of *Shigella* isolates by the disc-diffusion method, 2001-2002 (n=266)

Antimicrobial agent	Percentage of resistance (intermediate) among *Shigella* species				
	*S. boydii* (n=52)	*S. dysenteriae* (n=35)	*S. fexneri* (n=136)	*S. sonnei* (n=43)	Total (n=266)
Ampicillin	39	88 (7)	78	16	56 (2)
Amoxicillin/clavulanate	0 (17)	(37)	0 (42)	5)	0 (26)
Trimethoprim- sulphamethoxazole	42 (8)	90	68 (5)	87	72 (3)
Mecillinam	0 (0.5)	9 (3)	1 (1.5)	0	3 (3)
Nalidixic acid	33 (3)	98	18 (23)	54	51 (7)
Ciprofloxacin	0 (17)	0 (2)	0 (3)	0 (30)	0 (12)
Ceftriaxone/cefxime	3	0	0	5	2
Azithromycin	8 (78)	10 (73)	8 (38)	38 (60)	16 (62)
Tetracycline	58	90	85 (3)	78	79 (1)
Chloramphenicol	11	77	65	8	42
Erythromycin	100	98 (2)	93 (7)	100	97 (3)
Levofloxacin	0	0	0	0	0
Moxifloxacin	0	0	0	0	0
Rifampicin	100	100	100	100	100
Streptomycin	81 (19)	76	85 (15)	81	81 (8)
Cephalothin	19	0 (49)	0	41	14 (13)
Gentamicin	0	29	0	0	4

Figures in parentheses indicate percentages of strains exhibiting intermediate in resistance

Overall, MDR strains defined as simultaneously resistant to ≥3 of eight useful antimicrobial agents (ampicillin, trimethoprim-sulphamethoxazole, nalidixic acid, ciprofloxacin, mecillinam, tetracycline, azithromycin, and ceftriaxone/cefixime) were detected in 63% of the isolates (Table 2). It was significantly high (94%, p<0.01) in S. dysenteriae, followed by S. sonnei (60%) and S. flexneri (58%) and was low in S. boydii (27%, p<0.01). Resistance to ampicillin, trimethoprim-sulphamethoxazole, nalidixic acid, and tetracycline was most frequent (48%), followed by resistance to ampicillin, trimethoprim-sulphamethoxazole, and tetracycline (R-type ApSXTTe) (18%), nalidixic acid, trimethoprim-sulphamethoxazole, and tetracycline (R-type NalSXTTe) (14%). Resistance to one and two drug(s) was 8% and 19% respectively. Only 26 (10%) isolates were susceptible to all eight drugs tested.

**Table 2:** Patterns of resistance∗ of *Shigella* isolates to antimicrobial agents (n=266) in 2001-2002

Resistance to number of drugs, number of strains (%)	Resistance phenotype	
	Type	No. of strains
Resistance to >3 drugs, MDRstrains, 167 (63)		
Resistance to 6 drugs, 3	AAzCrNSXTT	2
	ACrMNSXTT	1
Resistance to 5 drugs, 6	AAzCrNSXT	1
	AMNSXTT	3
	AAzNSXTT	2
Resistance to 4 drugs, 86	ANSXTT	80
	AzNSXTT	6
Resistance to 3 drugs, 72	ANT	5
	AzNT	2
	ASXTT	33
	AAcT	2
	AzSXTT	5
	NSXTT	24
Resistance to two drugs, 51 (19)		
	AT	11
	AzN	5
	AzT	2
	ASXT	2
	NT	2
	SXTT	29
Resistance to one drug, 22 (8)		
	Az	2
	N	6
	SXT	7
	T	7
Susceptible, 26 (10)	-	26

^∗^Strains exhibiting intermediate resistance were not included A=Ampicillin; Az=Azithromycin; C=Ciprofoxacin; Cr=Ceftriaxone; M=Mecillinam; MDR=Multidrug-resistant phenotype; N=Nalidixic acid; SXT=Trimethoprim-sulphamethoxazole; T=Tetracycline

We compared resistance frequencies of *Shigella* isolates of 2001-2002 with those isolated during 1991-1992 (Table 3), which constituted part of our previous report (11). Resistance increased during the 2000s. Of note, the resistance to trimethoprim-sulphamethoxazole increased from 52% to 72% (p<0.01), resistance to nalidixic acid from 19% to 51% (p<0.01), and mecillinam from 0.5% to 3% (p<0.01). Resistance to ampicillin, tetracycline, and chloramphenicol was high (range 49-79%) in 1991-1992 and remained almost unchanged in 2001-2002 (Table 2). Strains with MDR phenotype increased to 63% in 2001-2002 from 52% (p<0.007) in 1991-1992.

**Table 3:** Comparison of antimicrobial resistance∗ of *Shigella* isolates to 12 antimicrobial agents by the disc-diffusion method between 1991-1992 and 2001-2002

Antimicrobial agent	Resistance rate (%) of *Shigella* isolates		
	1991-1992 (n=369)	2001-2002 (n=266)	p value
Trimethoprim-sulphamethoxazole	52	72	<0.01
Nalidixic acid	19	51	<0.001
Mecillinam	0.5	3	<0.001
Ampicillin	53	56	NS
Azithromycin	NT	16	ND
Tetracycline	74	79	NS
Ceftriaxone	0	2	ND
Cefxime	NT	2	ND
Amoxicillin-clavulanate	NT	0	ND
Chloramphenicol	49	42	NS
Gentamicin	0.2	4	<0.01
Ciprofoxacin	0	0	NS

^∗^Strains exhibiting intermediate resistance were not included ND=Not done; NS=Not significant; NT=Not tested

The MIC results of 154 available isolates (2001-2002) to nalidixic acid, ciprofloxacin, levofloxacin, azithromycin, and ceftriaxone are shown in the Figure. High modal MICs and MIC_90_ of nalidixic acid (modal MIC 128 μg/mL and MIC_90_ 256 μg/mL) and azithromycin (modal MIC of 6 μg/mL and MIC_90_ 8 μg/mL) and very low modal MIC (0.047 μg/mL) and MIC_90_ (0.023 μg/mL) of ceftriaxone were observed.

**Fig.**
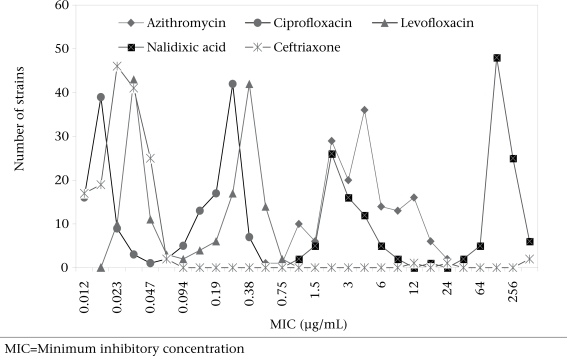
Modal minimum inhibitory concentrations of some useful antimicrobial agents among Shigella isolates (n=154) in 2001-2002, showing no significant increase of MIC to ceftriaxone

Of four *Shigella* isolates that were resistant to third-generation cephalosporins (TGC), two isolates of S. sonnei were highly resistant to ceftriaxone (MIC >256 μg/mL), and S. flexneri and S. boydii were intermediate to it (MIC 24 μg/mL). However, all but one ceftriaxone-resistant strain was susceptible to mecillinam.

MIC of ciprofloxacin-differentiated isolates having high MICs (0.064-0.38 μg/mL) and MIC_90_ (0.25 μg/mL) exhibiting decreased susceptibility to ciprofloxacin and nalidixic acid resistance (MIC ≥32 μg/mL) from nalidixic acid-susceptible (MIC <32 μg/mL) and ciprofloxacin-susceptible isolates having 10-fold lower MIC_90_ (MIC 0.023 μg/mL) and low MIC (range 0.012-0.047 μg/mL, Table 4). Levofloxacin showed similar results as ciprofloxacin. Of 154 *Shigella* isolates, decreased susceptibility to ciprofloxacin and resistance to nalidixic acid were detected among 110 (71%) strains. All isolates having decreased susceptibility to ciprofloxacin by MIC were susceptible to ciprofloxacin by the disc-diffusion method and resistant to nalidixic acid by the MIC and disc-diffusion method. Of 105 MDR strains tested, 91% exhibited decreased susceptibility to ciprofloxacin compared to 29% of 49 non-MDR strains (relative risk=3.20, p<0.00001).

**Table 4:** Correlation of results of the disc-diffusion method with that of ciprofoxacin MICs in *Shigella* isolates (n=154)

No. of strains	Disc-diffusion results		Ranee of MIC (∗MIC_90_) μg/mL	
	Nalidixic acid (30 μg)	Ciprofoxacin (1 μg)	Nalidixic acid	Ciprofoxacin
44	Susceptible	Susceptible	l-<32 (8)	0.012-0.047 (0.023)
110	Resistant	Susceptible	32->256 (256)	0.064-0.38 (0.25)

^∗^MIC_90_ was calculated separately for 44 nalidixic acid-susceptible and 110 nalidixic acid-resistant strains; MIC=Minimum inhibitory concentration

Three (two S. sonnei and one S. boydii) of four TGC-resistant strains were susceptible to amoxicillin-clavulanate and positive in DDST, indicating production of a class A ESBL (19), as described earlier (22). We were able to transfer ß-lactam resistance and ESBL production of these TGC-resistant strains to E. coli and *Shigella* by conjugation, which was encoded by a 50-MDa autotrasferable R-plasmid. The fourth isolate (S. flexneri) was resistant to amoxicillin-clavulanate but negative in DDST, thus exhibiting a class C (Amp C) ß-lactamase phenotype (19) mediated by a 94-MDa autotrasferable R-plasmid (22).

## DISCUSSION

*Shigella* causes invasive infection of the intestine that presents the most pressing challenge for providing effective antimicrobial therapy. Due to the emergence of resistance, antimicrobial agents, such as sulphonamides, tetracycline, ampicillin, trimethoprim-sulphamethoxazole, nalidixic acid, and mecillinam have all in succession been used as first-line drugs in Bangladesh and many countries of the world (2,7,12–14,23). During the past several decades, the organisms have progressively become resistant to most useful and inexpensive antimicrobial agents (7,8,11). Our study demonstrates an increasing incidence and spectrum of antimicrobial resistance of *Shigella* isolates in Bangladesh in 2001-2002. Rates of resistance to ampicillin, trimethoprim-sulphamethoxazole, and nalidixic acid increased to more than 50%. The resistance to tetracycline and chloramphenicol, which are not used currently for treating shigellosis, remained high and unchanged during the last decade. It is likely that the resistance rates observed in our hospital-based microbiology laboratory reflect the prevalence of resistance that exists in the community since nearly all cases of shigellosis were community-acquired, and the cultures were obtained on the day of admission. By analyzing the trends in the resistance patterns of various *Shigella* spp., we found that S. dysenteriae was at present significantly more resistant, followed by S. flexneri and other *Shigella* spp., in Bangladesh, especially to commonly-used antimicrobial agents. This finding is of special importance because S. flexneri is at present the predominant species in Bangladesh, like many other developing countries.

At present, pivmecillinam (oral form of mecillinam), fluoroquinolones, azithromycin, and third-generation cephalosporins (cefixime) are used in many countries for treating shigellosis caused by *Shigella* resistant to all first-line drugs. Pivmecillinam is currently used as an empirical antimicrobial therapy for shigellosis in Bangladesh with caution since resistance to it is emerging. The rate of mecillinam resistance, observed in the present study, is significantly lower than that observed in our earlier study in Bangladesh (11). This was due to lack of precise guidelines for determining susceptibility to mecillinam in the past that inherently overestimated the resistance rate. We used the CLSI (formerly NCCLS) methodology (24) for the determination of mecillinam susceptibility that reflects the true resistance rate in the present study. Azithromycin was found to be effective in treating shigellosis both in children and adults, including multidrug-resistant *Shigella*-associated infections (6,13). High modal MICs (6 μg/mL) and MIC_90_ (8 μg/mL) of azithromycin for *Shigella* isolates were observed in our study, and we detected in-vitro resistance to azithromycin in *Shigella* for the first time in Bangladesh. It is not clear why *Shigella* exhibits high modal MIC values to azithromycin. It is surprising that a significant proportion of isolates are resistant to this drug, although the drug is not commonly used for treating shigellosis in Bangladesh. However, the frequent use of macrolides for other infections and high carriage rate of *Shigella* in the gut of apparently healthy humans in Bangladesh might contribute to the emergence and spread of azithromycin-resistant *Shigella* strains (25). The significance of in-vitro resistance of *Shigella* to azithromycin is still unknown since intracellular concentration of azithromycin achieved in colonic cells and leucocytes exceeds serum concentration by 100-fold or more that could be fatal for intracellular bacteria (26). It should be mentioned that the two zones of inhibition produced by azithromycin by the disc-diffusion method and E-test sometimes caused difficulty in interpretation of results.

Quinolones are a good choice for the treatment of shigellosis in adults (3,13). Nalidixic acid was effective (23) and approved for use in the treatment of shigellosis in children aged less than three months. However, >50% of our *Shigella* isolates were resistant to nalidixic acid like many other developing countries (2,3,6,8). Fluoroquinolones (ciprofloxacin and norfloxacin) are also effective in treating nalidixic acid-resistant shigellosis, but paediatric use is limited by concerns about arthopathy and chondrotoxicity. However, reported data suggest that they are generally safe for the treatment of shigellosis in children (3,27). In 1994, S. dysenteriae type 1, resistant to nalidixic acid having decreased susceptibility to ciprofloxacin (MIC >0.125 μg/mL), was reported for the first time in Bangladesh by our group (9). The isolation rate of such strains of *Shigella* remarkably increased to 71% in 2001-2002 involving all species. The importance of strains having decreased susceptibility to fluoroquinolones was revealed recently by an outbreak of S. dysenteriae type 1 in 2002 in eastern India that affected 1,728 persons (attack rate of 25.6%), resulting in 16 deaths (28). Suboptimal clinical responses, therapeutic and microbiologic (positive culture after therapy) failures were associated with decreased ciprofloxacin susceptibilities of clinical isolates of Salmonella spp. and other bacteria in many countries, including India, the UK, Denmark, and the USA (29–31). To reduce such risks for humans, a recommendation has recently been made to lower breakpoint to 0.125 μg/mL for fluoroquinolones for Salmonella (29). With the increasing prevalence of *Shigella* strains having decreased susceptibility to fluoroquinolones such as ciprofloxacin, there is a need for careful observation of the outcome of ciprofloxacin therapy for shigellosis to detect suboptimal clinical response or therapeutic failures, if any. However, the problem is that the strains having decreased susceptibility to ciprofloxacin are not reported as these appear susceptible when subjected to ciprofloxacin-susceptibility testing (disc-diffusion method or by current MIC breakpoints) by the CLSI guidelines. As suggested by our study, resistance to nalidixic acid appears to be a useful screening marker for decreased ciprofloxacin susceptibility. Hence, future studies should evaluate the clinical outcome of the treatment of shigellosis caused by strains having susceptibility to ciprofloxacin but resistance to nalidixic acid. Further, in our earlier study, we detected a single-point mutation at codon Ser83 (TGC) to Tyr83 (TTC) in the quinolone resistance-determining region of gyrA gene of S. dysenteriae type 1, resulting in resistance to nalidixic acid with decreased susceptibility to ciprofloxacin (9). Additional mutations in the same gene (codon 87) and/or parC (codons 80 and 84) are known to result in complete resistance to ciprofloxacin (15). Thus, the use of fluoroquinolones is likely to result in complete resistance in *Shigella* strains harbouring resistance to nalidixic acid by additional mutations that we have witnessed recently in Bangladesh and India (32–33). Interesting is the fact that the recent isolates of ciprofloxacin-resistant S. dysenteriae type 1 in Bangladesh and India appeared to originate by such mechanisms: a mutation in codon 87 of the gyrA and additional mutation in codon 80 of parC genes (32–33). Although R-plasmid-mediated quinolone resistance may occur on rare occasion (34), it is not unlikely that we will see more and more ciprofloxacin-resistant *Shigella*-associated infections in the near future.

Cefixime and ceftriaxone were active against 98% of our isolates in vitro, but there is some dispute regarding the clinical efficacy of cefixime in treating shigellosis (6,35). Recently, cefixime was found to be clinically effective in 78% of children with shigellosis, predominantly caused by S. flexneri (6). However, it is an re-assuring finding that no increase in the MIC of ceftriaxone was observed in susceptible *Shigella* strains, unlike for ciprofloxacin or azithromycin. On the contrary, ESBL-mediated TGC resistance in *Shigella* strains was detected for the first time in Bangladesh. Detection of R-plasmid-mediated ESBL in *Shigella* isolates, transferable to E. coli K 12 and *Shigella* by conjugation, suggests that ESBL could spread resistance to third-generation cephalosporins among *Shigella* spp. and other pathogens in the community (22,36–37).

The limitation of our study is that we could not test all strains for antimicrobial susceptibility to detect exact rates of resistance to conventional and new useful antimicrobial agents. The isolates were from patients who came to the hospital for treatment or submitted faecal samples on the advice of physicians. Thus, strains might be associated with severe form of illnesses or from cases not responding to therapy reflecting high rates of resistance compared to those existing in the community. However, high rates of resistance among *Shigella* isolates have been reported in the community of Bangladesh (7,11–12).

Our study showed that *Shigella* strains developed resistance to many useful antimicrobial agents, including mecillinam, azithromycin, ceftriaxone, and cefixime in Bangladesh. Options for antimicrobial therapy for such MDR *Shigella*-associated infections are very limited leaving fluo-roquinoloes as the only option. Detection of decreased susceptibility to fluoroquinoloes in a high proportion of *Shigella* strains and complete fluoroquinolones-resistant S. dysenteriae type 1 (15–16,28,31–32) clearly demands careful and judicial use of these drugs to avoid rapid emergence and spread of resistance.

In conclusion, physicians should be aware of the high rates of antimicrobial resistance and increasing spectrum of resistance of Shigella spp. in Bangladesh. Continuous monitoring of the resistance patterns is essential, and antimicrobial susceptibility testing should be carried out on clinical isolates, and empirical antimicrobial therapy need to change accordingly. In addition, reduced susceptibility of *Shigella* strains to useful drugs should be identified by determination of MICs of antimicrobial agents for the early detection of the emergence of resistance. When indicated, pivmecillinam or ciprofoxacin might be considered for treating shigellosis with caution in Bangladesh.
